# Accurate Measurement of the True Plane-Wave Shielding Effectiveness of Thick Polymer Composite Materials via Rectangular Waveguides

**DOI:** 10.3390/polym11101603

**Published:** 2019-10-01

**Authors:** Robert Moučka, Stanislav Goňa, Michal Sedlačík

**Affiliations:** 1 Centre of Polymer Systems, University Institute, Tomas Bata University in Zlín, Trida T. Bati 5678, 760 01 Zlín, Czech Republic; moucka@utb.cz; 2 Faculty of Applied Informatics, Tomas Bata University in Zlín, Nad Stráněmi 4511, 760 05 Zlín, Czech Republic; gona@utb.cz

**Keywords:** electromagnetic shielding, waveguide, composite material, permittivity, permeability

## Abstract

This paper presents a methodology for accurately gauging the true plane wave shielding effectiveness of composite polymer materials via rectangular waveguides. Since the wave propagation of the waveguides is not in the form of plane wave patterns, it is necessary to post-process the S-parameters for the measured data of the waveguide lines to obtain such patterns and ascertain the effectiveness of true plane wave shielding. The authors propose two different methods to achieve this. The first applies simple renormalization of S-parameters, where reference impedance is changed from the value for the waveguide to that for free space, which ensures good accuracy of shielding effectiveness with a small degree of discontinuity across the range of frequencies. The other relies on rigorous extraction of the composite materials’ effective permittivity and permeability ascertained from rectangular waveguides; afterward, plane wave shielding effectiveness is calculated analytically and gives very high accuracy. Both procedures assume the given samples are isotropic in character. We validated the accuracy of the methodologies by conducting tests on a set of synthetic samples of 2 mm thickness with unit permittivity and variable conductivity and on a dielectric material of known permittivity (FR4 laminate). The applicability of both methods was further proven by analyzing the isotropic composite materials, a process involving the use of iron particles embedded in a dielectric matrix. The synthetic samples and an FR4 material were tested to check the accuracy of the methods. Based on numerical studies and measurements, we concluded that materials with a shielding effectiveness of up to 25 dB could be measured at a maximum amplitude error of 1 dB to 3dB to a frequency of 18 GHz, depending on the relative permittivity of the material; hence, the first method was suitable for approximation purposes. For maximal accuracy, the second method typically demonstrated an amplitude error of below 0.5 dB to the same frequency across the entire range.

## 1. Introduction

Measurement of plane wave shielding effectiveness is done on the wall of a metallic chamber, the given sample being illuminated by a plane wave generated by an excitation horn antenna; in accordance with a procedure described by the standards IEEE Std 285 and IEEE Std 299 [1,2]. In order to facilitate measurement of true plane wave shielding effectiveness, the size of the tested samples had to be at least a multiple of 3 to 5 times the figure for the free-space wavelength. This requirement limits the minimum measurement frequency for the given size of samples. According to IEEE Std 285, samples of up to 2 meters in size are necessary, enabling measurement down to the level of 750 MHz. However, in practice, far smaller samples are tested, usually ranging 600 to 300 mm in length. Consequently, the minimum frequency is limited to 2.5 GHz or 5 GHz, respectively.

Three factors restrict the measurement of the shielding effectiveness on transmission lines (see References [3,4,5,6,7,8]), thereby causing incompatibility with a set-up to research the same in free space. Firstly, the pattern of the electric field does not correspond with a plane wave. Secondly, the measurement line might exhibit dispersion behavior and non-orthogonal incidence of the wave. Thirdly, if testing the shielding effectiveness of coaxial lines, care must be taken to ensure higher-order modes are not excited inside the given sample [8]. It is necessary to take these three factors into account when processing S-parameter data obtained from measuring samples in waveguides and coaxial lines.

In this paper, two methodologies were applied to measure the shielding effectiveness of composite samples via rectangular waveguides.

The first of these was based upon simple renormalization of S-parameters valid for the waveguide to S-parameters valid for free-space, which is achieved through changing the reference impedance of the measured system. This novel approach ensures moderate accuracy in calculating shielding effectiveness with little discontinuity between the frequency bands for non-magnetic materials. The method’s exact level of precision is dependent on the value of the shielding effectiveness of the sample and its thickness. Part 2 of the paper contains a detailed discussion of such accuracy for a synthetic sample with a thickness of 2 mm, permittivity equal to 1 and 10 and variable conductivity.

Several papers in the literature on measuring shielding effectiveness by means of waveguides (with samples either placed inside or on a flange on the waveguide) have not investigated the aspect of renormalization. Hence, discontinuity is evident in their findings of shielding effectiveness during the transition between waveguide bands. For instance, a paper [8] describes gauging the effectiveness of thin composite materials at the relatively high figure of 50 to 60 dB. Moreover (see References [9]), the data reported exhibits an amplitude error between the WR90 and WR62 waveguide bands of approximately 2.5 dB. Therefore, the approach we describe herein that utilizes renormalization, together with an investigation of associated error in amplitude, is completely novel.

The measurement error of shielding effectiveness for the approximate method based on renormalization depends on five variables. These comprise electric and magnetic conductivity, relative permittivity and permeability, and the thickness of the measured material. Behavior of the error of shielding effectiveness was studied in detail for non-magnetic materials and it was observed that for the simple renormalization, the amplitude error of shielding effectiveness is less than 3 dB for 2 mm thick composite having a permittivity of 10 and an electrical thickness 0.3 *λ*_d_ up to 18 GHz. For non-magnetic 2 mm thick materials having a lower value of the relative permittivity, the error of the shielding effectiveness is reduced.

The second variant of the method relies on the rigorous extraction of the effective permittivity and permeability of the composite material, measured via rectangular waveguides [10,11,12,13], in adherence to the NRW (Nicolson–Ross–Wier) procedure. Subsequently, the effectiveness of plane wave shielding was calculated analytically. Others have applied this procedure in their research previously [14,15], reporting results for non-magnetic composites within the framework of a HIRF project [14]. Our contribution comprises a description of how to extract parameters for a metamaterial (a magnetic composite with iron particles) through utilizing waveguides, which we compare with the novel method of simple renormalization. In fact, the shielding properties of magnetic materials with iron particles have been written about recently [16] but the paper investigates the material properties of magnetic materials in coaxial test fixture.

Based on our experience of gauging complex permittivity and permeability in various transmission lines through the application of different extraction algorithms, we estimate an amplitude error of 0.5 dB for frequencies of up to 18 GHz in measuring shielding effectiveness via the second method. Besides determining the effective permittivity and permeability by the NRW procedure, these parameters can be obtained by optimization [15,17].

Both methods assume the given sample is isotropic in character.

## 2. Method of Measurement

In order to consider the effects of the waveguide’s dispersion and the different pattern of the electric field (compared to a plane wave in free space), a description is given in the next two subsections of a methodology, we have devised to achieve this end.

### 2.1. Measurement via a Rectangular Waveguide Based on Renormalization of Impedance

**Figure 1:**
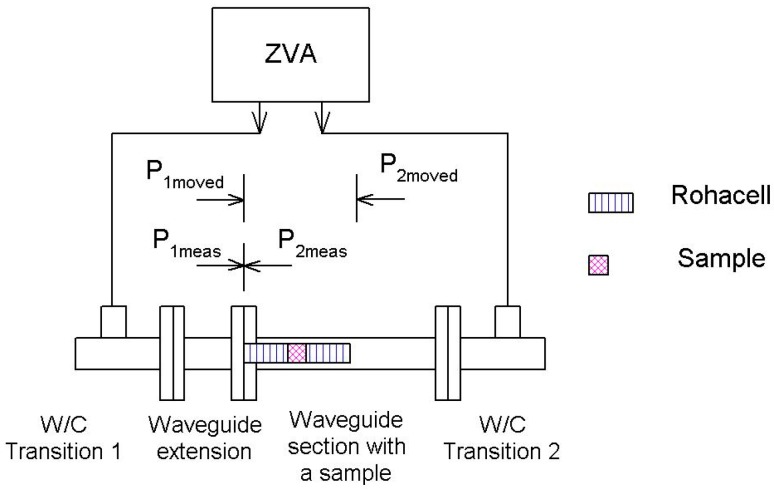
Set-up for measuring the shielding effectiveness of thick samples placed inside the rectangular waveguide.

Figure 1 illustrates our set-up for determining the shielding efficiency of thick samples by applying a rectangular waveguide. It consists of a vector network analyzer (an Agilent PNA-L-N5230A unit), 2 waveguide–to-coaxial transitions and 2 straight waveguide sections. Of the latter, the first section acts as an extension to the waveguide, thereby ensuring the first waveguide to coaxial transition is distanced from the reference plane. The sample under test is placed at the opening of the second waveguide section. Although it would be more advantageous to have two waveguide sections of identical length, these were not available to us in the laboratory. After performing a TOSM calibration for the waveguide, the S-parameters obtained were post-processed to shift the original reference planes—*P*_1meas_ and *P*_2meas_—into new respective positions—*P*_1moved_ and *P*_2moved_. The sample holder was made from Rohacell 71 HF.

The de-embedding process, which removes the effect of the Rohacell sample holder, is described by Equations (1) to (12). Firstly, the S-parameters of the assembly (holder + sample) were measured at the reference planes *P*_1meas_ and *P*_2meas_. Subsequently, these S-parameters were moved to the reference planes *P*_1moved_ and *P*_2moved_. Discussion on the procedure for shifting the reference planes is not included in this paper because it is merely a standard process for permitting measurement inside rectangular waveguides with a VNA. In our experiments, the shift in reference planes was performed at Matlab. The offset distance for port 1 was *l*_ofs1_ = 0, while for port 2 it equaled *l*_ofs2_ = 2*l* + *d*, where *d* represents the thickness of the sample and *l* is the length of the sample holder. The resulting S-parameters of the assembly (valid for planes *P*_1moved_ and *P*_2moved_) are given in Equation (1) below:

(1) $$S_{a}=S_{total}=\left(\begin{array}{cc} S_{11a} & S_{12a}\\ S_{21a} & S_{22a} \end{array}\right)$$

The S-parameters of the assembly were then converted into *ABCD* parameters by Equation (2), [18]:

(2) $$A_{total}=\frac{1}{S_{21a}}\left[\begin{array}{cc} \left(1+S_{11a}\right)\left(1-S_{22a}\right)+S_{12a}S_{21a} & \left(\left(1+S_{11a}\right)\left(1+S_{22a}\right)-S_{12a}S_{21a}\right)Z_{wg}\\ \left(\left(1-S_{11a}\right)\left(1-S_{22a}\right)-S_{12a}S_{21a}\right)/Z_{wg} & \left(1-S_{11a}\right)\left(1+S_{22a}\right)+S_{12a}S_{21a} \end{array}\right]$$

where *Z_wg_* is the field impedance of the rectangular waveguide, *γ_wg_* is the propagation constant in the waveguide and *l* is the length of the sample holder. Expressions for *Z_wg_* and *γ_wg_* are given by Equations (3)–(6).

(3) $$Z_{wg}=\frac{Z_{0}/\sqrt{\varepsilon_{r}}}{\sqrt{1-{\left(f_{c}/f\right)}^{2}}}$$

(4) $$\gamma_{wg}=\alpha_{wg}+j\beta_{wg}$$

(5) $$\propto_{wg}=\frac{-imag\left(k\right)}{\sqrt{1-{\left(f_{c}/f\right)}^{2}}}$$

(6) $$\beta_{wg}=\frac{2\pi }{\lambda_{0}}\sqrt{\varepsilon_{r}}\frac{1}{\sqrt{1-{\left(\frac{f_{c}}{f}\right)}^{2}}}$$

where *ε_r_* is the relative permittivity of the material placed inside a waveguide (in our case *ε_r_* = 1 or *ε_r_* = *ε_rholder_*). The symbol *Z*_0_ stands for the impedance of free space (*Z*_0_ = 120πΩ) and *k* represents the complex wave vector of the material placed inside the waveguide. Assuming the sample holder is non-magnetic, the wave vector *k* can be written as:

(7) $$k=\sqrt{-j\omega \mu_{0}\left(\sigma +j\omega \varepsilon_{0}\varepsilon_{r}\right)}$$

where *σ* is the conductivity of the sample holder and *ε*_0_ and *μ*_0_ represent the permittivity and permeability of free space.

The conductivity *σ* of the sample holder can be calculated by Expression (8):

(8) $$\sigma =\omega \varepsilon_{0}\varepsilon_{r}\tan\delta \;$$

where tan *δ* represents the loss factor of the sample holder.

The critical frequency *f*_c_ of the rectangular waveguide is calculated by Equation (9)

(9) $$f_{c}=\frac{c}{2\;a_{w}\sqrt{\varepsilon_{r}}}$$

When calculating values for matrix *A_t_* according to Equation (2), it is necessary to use the correct impedance which reflects the real value for permittivity *ε_r_* of the given material in the waveguide. Herein, the latter was filled with air, so the amount for *Z_wg_*_0_ was employed in Equation (2). This Equation effectively functions as a conversion formula between the *S* and *A* parameters and is widely recognized in transmission line theory [18].

The *ABCD* matrix of the sample is obtained via:

(10) $$A_{sample}=A_{holder}^{-1}A_{total}A_{holder}^{-1}$$

where *A_holder_* is the *ABCD* matrix for the sample holder, and *A_total_* is the *ABCD* matrix of the assembly, consisting of the sample and two sample holders. The *ABCD* matrix of the sample holder is given by an analytical expression from theory on transmission lines [18].

(11) $$A_{holder}=\left[\begin{array}{cc} \cos h\left(\gamma_{wg}l\right) & Z_{wg\;}\sin h\left(\gamma_{wg}l\right)\\ \frac{1}{Z_{wg}}\sin h\left(\gamma_{wg}l\right) & \cos h\left(\gamma_{wg}l\right) \end{array}\right]$$

The propagation constant *γ_wg_* and impedance *Z_wg_* are calculated according to Equations (3) and (4), with the assumption that *ε_r_* = *ε_r_*_h_, where *ε_r_*_h_ is the permittivity of the Rohacell sample holder. Note, for Rohacell HF71, the measured value *ε_r_*_h_ = 1.11 was applied. Measurement was performed in the laboratory in the WR90 band by employing a propagation phase method. The manufacturer states a permittivity value for Rohacell of approximately 1.09 at 10 GHz. Since losses of Rohacell HF 71 are not high and exert no influence on the de-embedding process, the constant value for such a loss factor tan *δ* equals 0.004 was assumed for all frequencies in our experiments; in fact, this is the measured value usually specified by the manufacturer at 10 GHz.

Finally, the S-parameters of the sample (normalized to impedance *Z_wg_*_0_) are obtained by:

(12) $$S=\left[\begin{array}{cc} \frac{A+B-C-D}{\Delta } & \frac{2\left(AD-BC\right)}{\Delta }\\ \frac{2}{\Delta } & \frac{\left(-A+B-C+D\right)}{\Delta } \end{array}\right]$$

where *A* = *A_sample_*, *B* = *A_sample_*/*Z_wg_*_0_, *C* = *A_sample_ Z_wg_*_0_, *D* = *A_sample_* and symbol Δ is given by the expression Δ = *A* + *B* + *C* + *D*; impedance *Z_wg_*_0_ stands for the impedance of the air-filled waveguide according to Equation (3) and assuming *ε_r_* = 1.

After de-embedding, renormalization is performed with respect to the free-space impedance *Z*_0_. Initially, renormalization converts the S-parameters for the sample *S_sample_* into *A* parameters at impedance *Z_wg_* according to Equation (3), wherein *ε_r_* = 1. Afterward, the A-matrix is converted back into S-parameters by Equation (12), assuming the value of impedance *Z*_0_ = 377 Ω.

**Figure 2:**
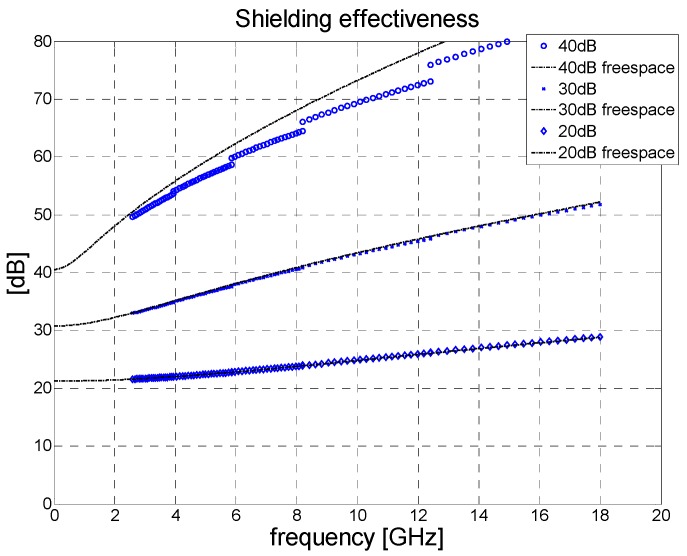
Comparison of values for true plane-wave shielding effectiveness obtained by measuring the material in the rectangular waveguide followed by the renormalization process.

We tested the accuracy of the proposed methodology by conducting a set of simulations in the Full-Wave Simulation software (CST microwave studio, FD solver) for the architecture detailed in Figure 1 pertaining to the given sample (2 mm in thickness). The sample demonstrated a permittivity of *ε_r_* = 1 and conductivity equal to 280, 89, or 28 S/m. These values for conductivity resulted in the free-space shielding effectiveness of 40, 30, and 20 dB, respectively. The shielding effectiveness which had been computed was compared to that obtained by the same simulation software in free space and valid for the plane wave (Figure 2).

**Figure 3:**
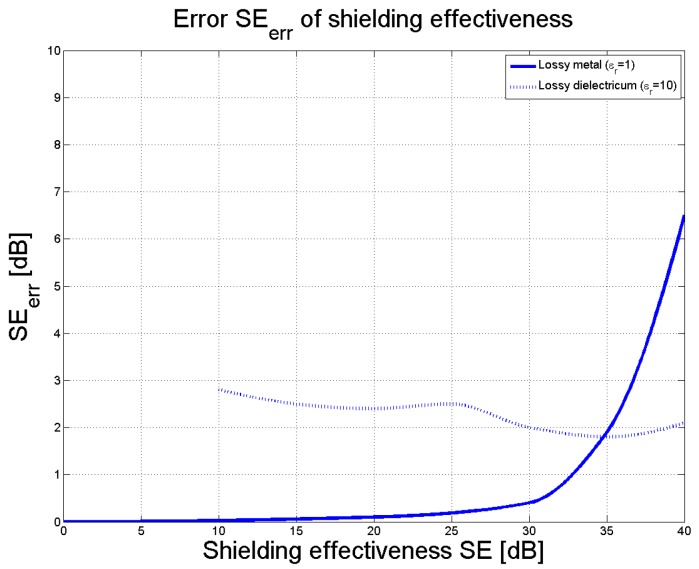
Dependence of the SE error for an increase in SE values.

It was found that the 2 mm thick sample of lossy metal (*ε_r_* = 1) exhibited shielding effectiveness that ranged from 0 to 40 dB; dependence was evident in the amplitude error *ERR*_SE_ between the shielding effectiveness obtained in the waveguide and the shielding effectiveness in free space, as visible in Figure 3. The graph reveals that for a given maximum tolerated error of *ERR*_SE_ = 1dB the theoretical maximum shielding effectiveness that can be measured in the waveguide by the approximate method is circa 32 dB. For larger values of SE, the error increases very rapidly. We estimated a practical limit of 25 dB due to the air gap between the sample and wall of the waveguide.

If the sample possesses a greater dielectric constant than the approximated value, the error exceeds that of a lossy metal. For example, for *ε_r_* = 10, the maximum error of shielding effectiveness remains below 3 dB for all frequencies up to 18 GHz (Figure 3, dotted line).

The measurement error of shielding effectiveness for magnetic materials has not been studied in detail in the case of the approximate method. However, a composite system similar to ours (effective permittivity 6 and effective permeability 1.2) was investigated [14]. Our simulations showed that the approximate method is not suitable for this case since the measurement error of shielding effectiveness is too large. Also, the maximal value of shielding effectiveness (dynamic range), which can be measured for magnetic materials, is low. Nevertheless, our weakly magnetic composite material having an SE of approximately 4.5 dB at 18 GHz could be measured with good accuracy of about 1 dB.

### 2.2. Measurement Inside a Rectangular Waveguide Based on Effective Parameters for the Given Material

The set-up for measurement was identical to that detailed in the previous section (see Figure 1), differing only in how the S-parameters were processed. Following a procedure stated in the literature [13], the complex permittivity and permeability of the sample inside the rectangular waveguide were arrived at by Equations (13) to (19), as follows:

(13) $$K=\frac{S_{11}^{2}-S_{21}^{2}+1}{2S_{11}}$$

where *K* is a factor stipulated in the standard NWR technique, as corroborated in the reference [13].

(14) $$\Gamma =K\pm \sqrt{K^{2}-1}$$

*Γ* constitutes the reflection coefficient at the interface between the air and the measured material. The sign given in Equation (14) is selected appropriately to obtain |Γ|<1.

Afterward, the wave propagation term *T* (propagator) is calculated according to the equation below:

(15) $$T=\frac{S_{11}+S_{21}-\Gamma }{1-(S_{11}-S_{21})\Gamma }$$

(16) $$\frac{1}{\Lambda^{2}}=-{\left(\frac{1}{2\pi D}\left(\ln\left(\frac{1}{T}\right)+2\pi m\right)\right)}^{2}$$

where *D* represents the thickness of the sample in the waveguide and *m* constitutes an integer, as the complex logarithm is multivalued and has to be determined by comparing the measured and calculated group delay for the dielectric sample in the waveguide; more details on *m* can be found in the referenced study [13]. Herein, both the FR4 laminate and composite material were of limited electrical thickness; hence, coefficient *m* remained at naught.

In Equation (16), *Λ* can be calculated by working out the square root of the inverse of the same Equation:

(17) $$\Lambda =\sqrt{\frac{1}{\Lambda^{2}}}$$

where the square root in Equation (17) is chosen to facilitate a positive, imaginary portion.

Subsequently, effective permittivity and permeability are determined by the Equations (18) and (19) (see References [13]):

(18) $$\mu_{reff}=\frac{1+\Gamma }{(1-\Gamma )\Lambda \sqrt{{\left(\frac{1}{\lambda_{0}}\right)}^{2}-{\left(\frac{1}{\lambda_{c}}\right)}^{2}}}$$

(19) $$\varepsilon_{reff}=\frac{\lambda_{0}^{2}}{\mu_{reff}}\frac{1}{\left(\frac{1}{\lambda_{c}^{2}}+\frac{1}{\Lambda^{2}}\right)}$$

where *λ*_0_ and *λ_c_* represent the free-space wavelength and critical wavelength of the air-filled rectangular waveguide, respectively. The critical wavelength of the waveguide is given by the expression *λ_c_* = 2*a*_w_; free-space wavelength is derived by *λ*_0_ = *c*/*f*.

Equations (20)–(24) permit calculation of the true free-space reflection and transmission coefficient (TE polarization) of the layer, the latter pertaining to thickness *D* and permittivity and permeability *ε_r_* and *μ_r_*.

(20) $$\left[M_{TE}\right]=\left[\begin{array}{cccc} 1 & -cosh\left(\gamma_{1}D\right) & -sinh\left(\gamma_{1}D\right) & 0\\ 0 & 1 & 0 & -1\\ \gamma_{0}\mu_{r} & \gamma_{1}sinh\left(\gamma_{1}D\right) & \gamma_{1}cosh\left(\gamma_{1}D\right) & 0\\ 0 & 0 & \gamma_{1}/\gamma_{r} & -\gamma_{0} \end{array}\right]$$

(21) $$b=\left[-1;0;\gamma_{0}\mu_{r};0\right]$$

(22) $$x={\left[M_{TE}\right]}^{-1}\left[b\right]$$

(23) $$S_{11}=x\left(1,1\right)$$

(24) $$S_{21}=x\left(4,1\right)$$

where *γ*_0_ and *γ*_1_ stand for complex propagation constants in free space and in the material, respectively; *D* represents the thickness of the material; the constants *γ*_0_ and *γ*_1_ are worked out by Equations (25) to (30) below:

(25) $$k_{0}=\sqrt{-j\omega \mu_{0}\left(j\omega \varepsilon_{0}\right)}$$

(26) $$\gamma_{0}=\sqrt{\alpha^{2}+\beta^{2}-k_{0}^{2}}$$

(27) $$\gamma_{1}=\sqrt{\alpha^{2}+\beta^{2}-k_{1}^{2}}$$

(28) $$\alpha =k_{0}sin(\vartheta )cos(\varphi )$$

(29) $$\beta =k_{0}sin(\vartheta )sin(\varphi )$$

(30) $$k=\sqrt{-j\omega \mu_{0}\mu_{r}\left(j\omega \varepsilon_{0}\varepsilon_{r}\right)}$$

Therein, *ϑ* and *φ* constitute the angles of incidence of the plane wave; in our experiments, angles *ϑ* and *φ* were considered as nought.

Bringing this section to a close, we conclude that this method for calculating shielding effectiveness is more accurate than the method based on renormalization of impedance (Section 2.1), the same also permitting measurement of higher values for shielding effectiveness. However, in practice, the maximum value for shielding effectiveness that it is possible to gauge is constrained by the air gaps between the sample and wall of the waveguide. We estimate that the practical maximal value for SE is approximately 25 to 30 dB.

### 2.3. Preparation of the Polymer Composite Samples

**Figure 4:**
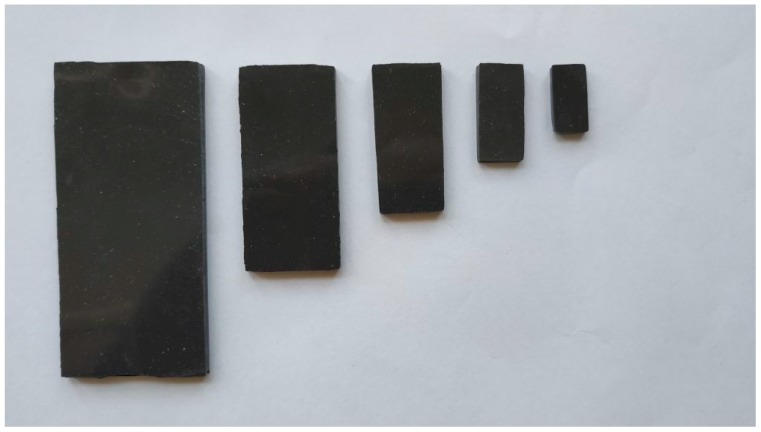
The set of prepared polymer composite samples for all 5 waveguides (from left: WR284, WR187, WR137, WR90, and WR62).

The polymer composite samples contained carbonyl iron particles (SL grade, BASF) at 10% concentration of volume, homogeneously distributed throughout by mixing the same in an elastomeric silicon-based polymer matrix (Sylgard 184, DowCorning). A mixture of the particles, silicon, and a curing agent was cast in a rectangular mold (100 × 100 mm; 2 mm thick) and cured for 2 h at 80 °C. Subsequently, a sample for each waveguide was cut out with a dedicated cutter, the dimensions of which reflected a cross-section of the given waveguide. Figure 4 shows the set of samples we used.

## 3. Results

Here we present a detailed description of our results, covering the reflection and transmission coefficients of the FR4 laminate and composite material. The first part is given over data obtained by the approximation method based on the renormalization of impedance, while the other summarizes those of the rigorous, accurate procedure utilizing the materials’ effective parameters.

### 3.1. Results for the Method of Renormalization of Impedance

In adherence to the methodology described in Section 2.1 and 2.2, measurement of the FR4 laminate and the composite material was performed in a set of rectangular waveguides, nos. WR284, 187, 137, 90, and 62. Samples were precisely positioned with the aid of Rohacell separators (Rohacell HF 71); the left edge of the cascade (holder + sample + holder) was always placed flush with the left flange of the waveguide in which the cascade was inserted. The lengths of the Rohacell sample holders differed in accordance with the individual bands, i.e., 33, 20, 14, 10, and 7 mm for the bands ranging from WR284 to WR62.

Prior to gauging the S-Parameters with the individual bands, we calibrated the VNA. For the lower bands (WR284, 187, and 137), TOSM calibration was used, whereas we utilized TRL for the higher ones of WR90 and WR62. Afterward, the measurement of the S-parameters was conducted and the Touchstone results then imported to Matlab.

**Figure 5:**
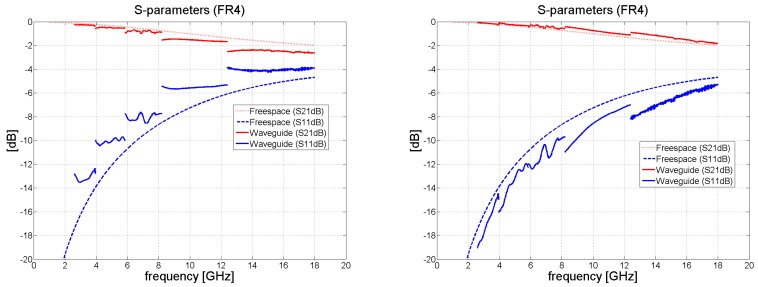
Magnitude of S_11_ and S_21_ in dB for FR4 (waveguide measurements de-embedded to remove the effect of the sample holder); (**left**) without re-normalization, S-parameters with respect to *Z_wg_*_0_; (**right**) with renormalization from impedance *Z_wg_*_0_ to free-space impedance *Z*_0_ = 377 Ω.

**Figure 6:**
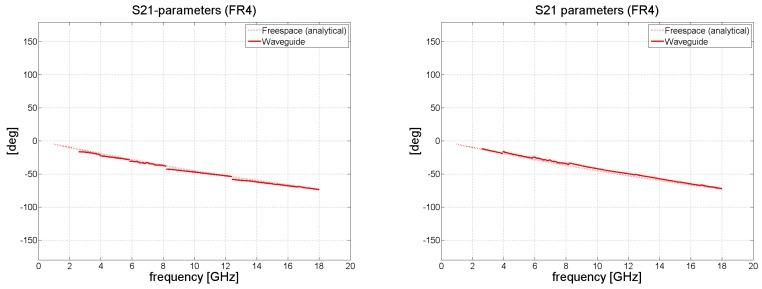
Phase shift of *S*_21_ in degrees for the FR4 laminate (waveguide measurements de-embedded); (**left**) without renormalization; (**right**) with renormalization to *Z*_0._

**Figure 7:**
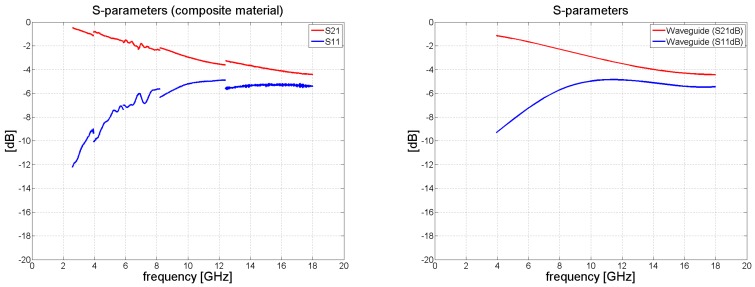
Magnitude of *S*_11_ and *S*_21_ in dB for the composite sample (waveguide measurements de-embedded and renormalized to free-space impedance *Z*_0_); (**left**) measurement in the bands WR284, 187, 137, 90, and 62; (**right**) smoothed data interpolated by a cubic spline.

**Figure 8:**
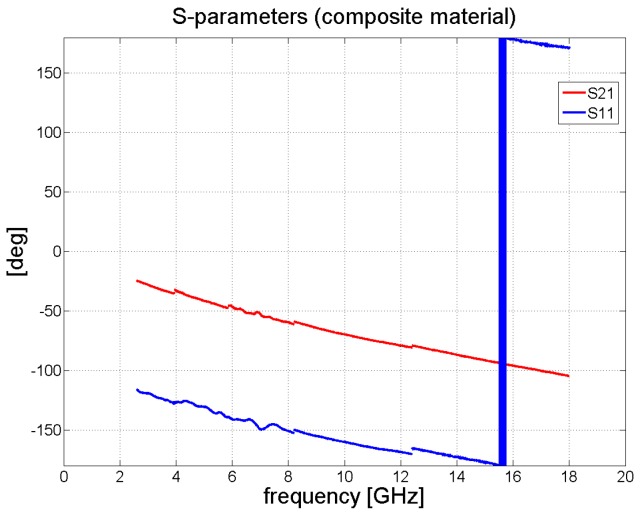
Phase of *S*_11_ and *S*_21_ in degrees for the composite sample.

Following this, the data were processed in Matlab, in accordance with the procedure described in Section 2.1. The computed results of the reflection coefficient *S*_11_ and transmission coefficient *S*_21_ for FR4 and the composite material are shown in Figure 5, Figure 6, Figure 7 and Figure 8. Very high accuracy was observed for FR4 between the measured transmission coefficient *S*_21_ and transmission coefficient predicted analytically; the maximum amplitude error for the highest frequency band was only 0.3 dB. The amplitude error for the reflection coefficient equaled approximately 2 dB for the highest frequency band. The extent of the amplitude error depends on the frequency and differs for each frequency band, as a consequence of the angle of the incidence also being influenced by frequency. For the purposes of analytical calculation, normal incidence was assumed. The maximum insertion loss for the 1.57 mm thick laminate FR4 occurred at 18 GHz, at the level of about 2 dB.

When measuring the composite material, the transmission coefficient *S*_21_ decreased monotonically with frequency to the value of circa –4 dB at 18 GHz. The results for *S*_21_ and *S*_11_ are summarized in Figure 7.

Figure 6 and Figure 8 illustrate the behavior of the phase of the transmission coefficient. In the case of FR4, a strong agreement was achieved between the measured and analytically calculated results.

### 3.2. Results for the Method Based on the Effective Parameters of the Material

Here we show our results obtained by the method based on the materials’ effective parameters—a rigorous approach for accurately determining free-space reflection and transmission coefficients after gauging the S-parameters in the rectangular waveguides.

**Figure 9:**
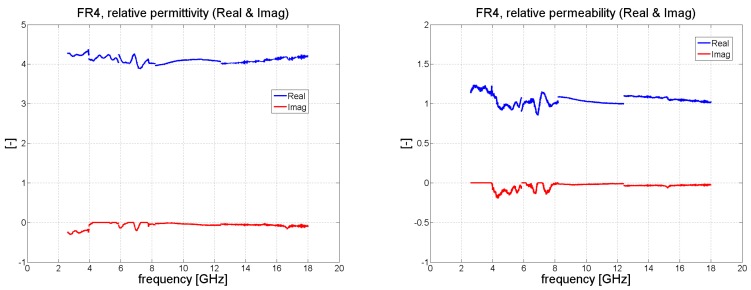
The effective material parameters of FR4 retrieved after measuring the S-parameters.

**Figure 10:**
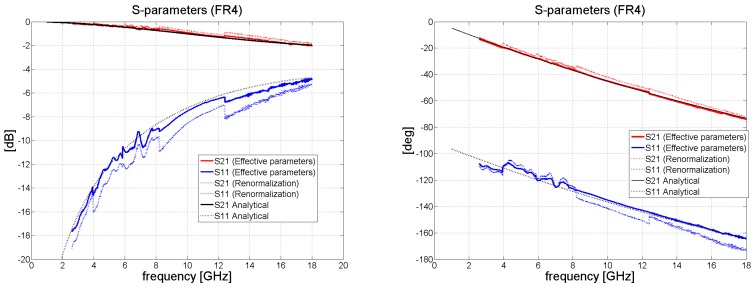
The true plane wave transmission and reflection coefficients for the FR4 laminate obtained by measuring the S-parameters and applying the accurate methodology.

**Figure 11:**
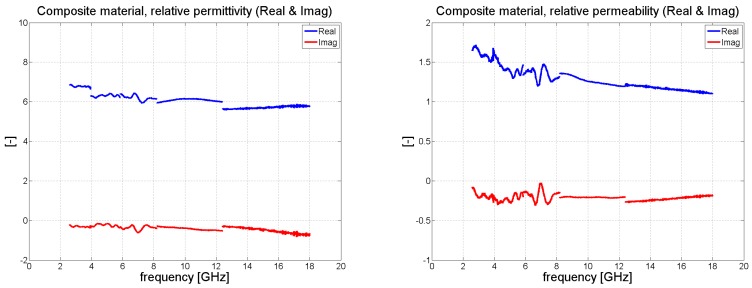
Retrieved effective permittivity and permeability of the composite material.

**Figure 12:**
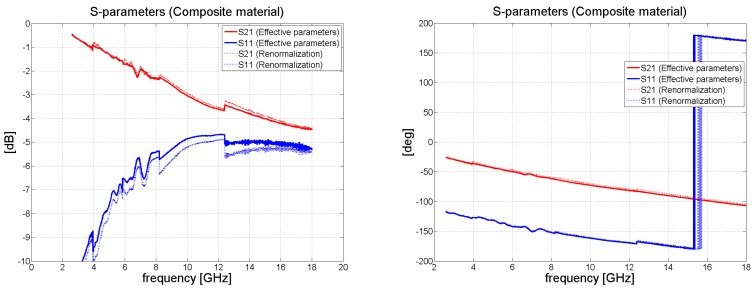
Comparison of the transmission and reflection coefficients for the composite material determined via the simple renormalization and effective material approaches.

Following the procedure explained in Section 2.2, processing the S-parameters obtained for the given composite material produced the results shown in Figure 9, Figure 10, Figure 11 and Figure 12. From Figure 9 it can be concluded that the real portion of the relative permittivity for the FR4 material is close to the value of 4.2. The relative permeability for the FR4 laminate should ideally have been 1 but errors in measurement gave rise to the values 1.1–1.2. Since the FR4 laminate was only 1.57 mm in thickness, overall dissipation within the sample was limited. As a consequence, it was not possible to discern the electric loss tangent of the material with any great accuracy. Some low positive values for the imaginary part of the relative permittivity and permeability equaled naught.

Taking into account complex relative permittivity and relative permeability, we set about calculating the free-space reflection and transmission coefficients, as shown in Figure 10. The transmission coefficient for the FR4 was very close to that predicted analytically, with a maximum error in the highest frequency band of approximately 0.2 dB. The typical error in the reflection coefficient was seen to be at about 0.5 dB for the WR90 and WR62 bands. Figure 10 compares the results obtained by the accurate method with those for the approximation approach, illustrating that the former outperforms the latter. In addition, the same figure presents the amplitude of reflection and transmission coefficients and phase behavior.

Measurement of the 2 mm thick composite samples was carried out in the same way as for the FR4 laminate. Figure 11 details the resulting values for complex permittivity and permeability. The effective permittivity of the composite material changed from 6.5 to 5.8 alongside an increase in frequency. Since the laminate contained iron particles, it also exhibited magnetic behavior, yet its value for effective permeability was quite low, varying from 1.7 to 1.1 monotonically with a rise in frequency. The resulting transmission and reflection coefficients for the composite sample were calculated analytically (see Figure 12); the same figure shows that the accurate method was in a strong agreement with the results for the approximation approach. Our findings are close to those reported in the literature [16], wherein a composite containing iron particles was measured in a coaxial transmission line; thereto, we also observed a very similar trend in the real portion of permittivity and permeability, as well as analogous numerical values for the same. The permittivity reported in the said study [16] varies between 7 to 5.5 for frequencies in the range of 2 to 18 GHz, while permeability alters from 2 to 1.2. Notably, the results disclosed in another paper [18] were obtained by Agilent material measurement software, while we gauged the properties of our composite material by the Matlab program used for measuring materials.

To conclude this section, it is worthy to mention that the shielding effectiveness of the material is calculated from the decibel value for transmission coefficient *S*_21_ through the act of changing its sign.

## 4. Conclusions

We have presented in this paper a novel approach for measuring free-space reflection and transmission coefficients for thick polymer samples placed in rectangular waveguides. The two variants proposed comprise an approximate one, based on simple renormalization of impedance, and an accurate one with effective modeling of the material. The accuracy of the methods was studied by using a synthetic lossy metal and FR4 laminate. The procedure we describe involved tests with a thick composite sample, consisting of a dielectric matrix and conductive iron particles. Our measurements and simulations show that the accurate method represents a precise approach for determining the true free-space shielding effectiveness of materials. The maximum error of measurement of shielding effectiveness is about 0.5 dB for the accurate method. By taking into account the effect of air gaps, the composites with a shielding effectiveness of up to 25 dB can be measured. Higher values of SE have to be measured via a flanged architecture, which is not discussed in this paper.

For the approximate method, the accuracy of measurement of shielding effectiveness was studied on non-magnetic material in detail. Its value is dependent on the value of the effective permittivity. Materials with effective permittivity of less than 10 have the error of shielding effectiveness less than 3 dB with an overall dynamic range of 25 dB. For lower values of permittivity the error of SE is reduced.
